# Serum Cytokine Levels and Their Relation to Clinical Features in Patients with Autoimmune Liver Diseases

**DOI:** 10.1155/2017/9829436

**Published:** 2017-02-19

**Authors:** Dilyara Akberova, Andrei P. Kiassov, Diana Abdulganieva

**Affiliations:** ^1^Kazan State Medical University, Kazan, Russia; ^2^Institute of Fundamental Medicine and Biology, Kazan Federal University, Kazan, Russia

## Abstract

Serum cytokine levels were explored in a combined group of patients with autoimmune liver diseases (AILDs) and separately in patients with autoimmune hepatitis (AIH) and overlap syndrome. Overall, 60 patients with AILD, among them 32 patients with AIH and 28 patients with overlap syndrome, were included in the cross-sectional study. Serum cytokine levels were measured at baseline and compared to those of 21 healthy controls. Patients with AILD had significantly higher levels of IL-6 (0.70 (range 0.17–99.86) in patients with AILD compared to 0.40 (range 0.14–2.65) in controls, *p* < 0.01), IL-8 (1.66 (0.45–34.58) versus 0.53 (0.35–2.38), resp., *p* < 0.01), and TNF-*α* (2.61 (0.23–120.88) versus 1.65 (0.21–7.54), resp., *p* < 0.01). Adjusted logistic regression analysis revealed a pronounced relation of IL-8 and AILD, 48.36 (3.63–643.60), as well as AIH, 18.54 (1.08–318.54), and overlap syndrome, 23.85 (2.37–240.23), while the associations between the level of other cytokines and AILD were assessed as nonsignificant. In the language of absolute numbers, the increase of IL-8 serum level by 1 pg/mL had increased the chance for a patient to find himself in a group of AILD by 48.36 times. Also, high IL-8 serum levels were strongly related to clinical parameters.

## 1. Introduction

Autoimmune liver diseases (AILDs) include a broad range of autoimmune disorders affecting the liver and biliary system, with primary biliary cirrhosis (PBC), primary sclerosing cholangitis (PSC), and autoimmune hepatitis (AIH) being the classic types of AILD. While AIH targets the hepatocytes and is mainly associated with hepatocellular injury, the targets of the autoimmune attack in PBC and PSC are the biliary epithelial cells, which leads to a predominance of cholestatic features [1, 2]. AIH and PBC are the most frequent AILDs with an incidence around 1-2 per 100000 population per year for each disease [3] and a prevalence of 2–40 cases per 100000 population in different parts of the world, with the highest incidence and prevalence found in the United States and northern Europe [4]. Conditions exhibiting features of 2 different AILDs are commonly designated as overlap syndromes. They are not uncommon, with a combination of PBC and AIH occurring in 2–19% of all patients with AIH, and usually show a progressive course towards cirrhosis and liver failure without adequate treatment [2].

Although the exact mechanisms of the AILDs are unclear [5], broadly similar pathogenic pathways of injury have been postulated for AIH, PBC, and PSC, and these comprise a combined influence of environmental triggers, genetic predisposition, and failure of immune tolerance mechanisms, which, in turn, collaborate to induce an antibody- and* T* cell-mediated immune attack against liver-specific targets, leading to a progressive necroinflammatory and fibrotic process in the liver [2]. Persistent liver injury, associated with chronic AILD, leads to persistent inflammation, cell proliferation, and the deposition of extracellular matrix proteins by hepatic stellate cells and portal myofibroblasts. Liver cirrhosis, and the resultant loss of normal liver function, inevitably ensues [1].

Although it is widely recognized that AILDs are mainly* T* cell mediated diseases [6], it is also shown that cytokines, such as interleukins (IL), play one of the key roles in the pathogenesis of the disease and in liver innate lymphoid cells and natural killer* T* cells activation [7]. The exact role of the certain cytokines and their utility as biomarkers for predicting disease outcomes, or as a diagnostic tool, as well as their potential use as a treatment target are yet to be explored, although monoclonal antibodies, for example, antibodies directed against IL-12/IL-23, are even now considered to be one of the potential treatment options for AILD [8].

The aim of the present study was to reveal the differences in the serum cytokine levels between patients with AIH and overlap syndrome, and between the combined group of patients with AILD and healthy controls, and to explore the associations between the cytokine levels and the clinical features of the disease in patients with AILD.

## 2. Patients and Methods

### 2.1. Patients

Overall, 60 patients with AILD (females, 54 (90%), mean age 48.2 ± 15.1 years), among them 32 patients with AIH (females, 28 (87.5%), mean age 43.2 ± 15.5 years) and 28 patients with overlap syndrome (females, 26 (92.8%), mean age 53.9 ± 12.6 years), were consecutively included in the cross-sectional noninterventional cohort study. Inclusion was performed in a single center (Department of Gastroenterology, Republican Clinical Hospital, Kazan, Russia) and took place between April 2015 and May 2016. The inclusion criteria were age of 18 years and above, diagnosis of AILD established according to the actual recommendations: PBC-EASL (2009) [9], AIH-AASLD (2010) [10], AIH-PBC overlap syndrome, IAIHG position statement (2011) [11], and patients' ability and willingness to provide the informed consent for the study. Patients were not included if they had any of the following: viral hepatitis B or C (serum markers or history), Wilson's disease, nonalcoholic steatohepatitis, alcoholic liver disease, drug-induced hepatitis, pregnancy, and any severe conditions or other conditions that would in the opinion of the study physician preclude participation in the study. Every patient, who was observed at the department with AILD and was satisfying the requirements of inclusion criteria, was invited to participate in the study.

As a control group, 21 healthy age- and sex-matched volunteers (females, 18 (85.7%), mean age 47.8 ± 10.6 years) were examined. All of them had an inconspicuous medical history without diagnosed active gastrointestinal diseases, infections, and liver diseases, no history of major intestinal surgery during the past 12 months or alcohol abuse, and no history of antibiotics or anti-inflammatory drugs intake within the last month. The aforementioned exclusion criteria were also applied to the controls. Most of the controls in this study were healthcare workers, who volunteered to be included in the control group.

### 2.2. Study Design

All patients underwent complete clinical and laboratory examination. Demographic data (such as disease duration, age, and gender), clinical manifestations, (such as presence and severity of ascites, portal hypotension, hepatic encephalopathy, and extrahepatic symptoms), and vital parameters of the patients were assessed on the day of inclusion. Ongoing and previously taken treatments and concurrent medications were recorded. Laboratory examinations included common blood count and biochemical blood tests, as well as immunological tests (antinuclear, antimitochondrial, antismooth muscle titers, levels of circulating immune complexes and gamma-globulins), according to standard clinical protocols.

### 2.3. Cytokine Analysis

Serum samples were collected at baseline from each enrolled subject. Serum cytokine levels were analyzed using 〈〈Bio-Plex 200〉〉 (Bio-Rad, Hercules, CA, USA) Multiplex Immunoassays following the manufacturer's instructions. Serum levels of seven cytokines were measured: IL-2, IL-4, IL-6, IL-8, IL-10, interferon-gamma (IFN-*γ*), and tumour necrosis factor-alpha (TNF-*α*). Median fluorescence intensities were measured using Luminex 200 analyzers (Luminex, Austin, TX, USA). Collected data were analyzed using with MasterPlex CT control software and MasterPlex QT analysis software (MiraBio, San Bruno, CA, USA). Standard curves for each analyte were generated using standards provided by manufacturer.

### 2.4. Statistical Analysis

Statistical processing and analysis of obtained data were performed by IBM SPSS Statistics version 23 (Chicago, IL, USA), Statistica version 12.5 (Statsoft), and Microsoft Excel 2013. The descriptive data are presented as median and range (for cytokine levels), or M ± SD, where M is the mean and SD is the standard deviation (for demographic and clinical data). The groups were compared using Mann–Whitney* U* test and Kruskal-Wallis test. Dichotomous parameters were compared between groups using the chi-square test. Obtained differences were considered statistically significant if *p* < 0.05. The relation between the chance for disease and serum level of cytokines was assessed using logistic regression analysis. Variables that were significant on univariate analysis were further analyzed using multivariate analysis to adjust for confounders, such as the level of other cytokines, age, and gender.

### 2.5. Ethics Committee Approval

The study protocol was approved by the local ethics committee of the Kazan State Medical University (protocol number 10, 23 December 2014). All patients signed the informed consent form before being included in the study.

## 3. Results

### 3.1. Clinical Characteristics of Patients

The clinical and laboratory data for the patients at baseline are shown in Table 1. There were some differences observed between the groups. Overall, patients with overlap syndrome, as compared to patients with AIH, had shorter duration of symptoms (4.7  ±  4.5 versus 6.9  ±  6.3 years, resp.) and higher levels of alanine aminotransferase (ALT) (147.8  ±  183.6 versus 68.8  ±  72.8 U/L) and alkaline phosphatase (337.8  ±  176.9 versus 107.7  ±  71.4 U/L), as well as higher erythrocyte sedimentation rates (ESR) (33.12  ±  13.3 versus 17.3  ±  12.1 mm/h), and took glucocorticoids less frequently (15 (53.6%) versus 25 (78.1%), resp.), although mean doses of corticosteroids were overall comparable (17.8  ±  15.5 versus 26.1  ±  6.2 mg/day, resp.).

### 3.2. Comparison of Serum Levels of Cytokines between the Patients with AILD and Controls

**Figure 1:**
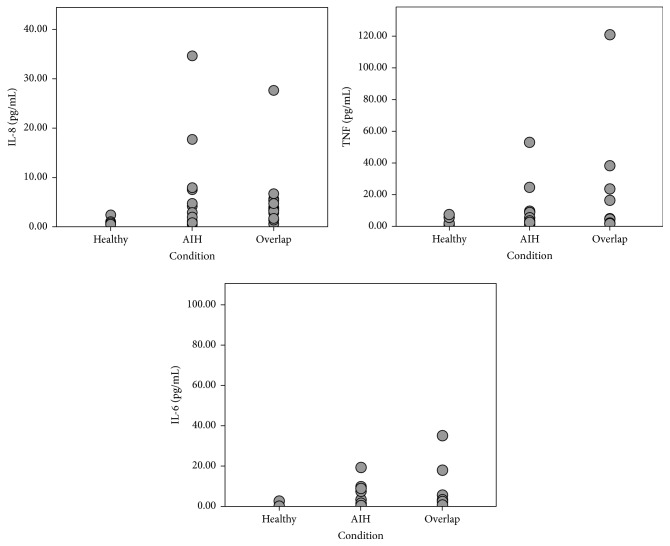
Circulating serum interleukin-8, tumour necrosis factor-*α* (TNF-*α*), and interleukin-6 concentrations in patients with autoimmune hepatitis (AIH) and overlap syndrome and in healthy controls. Differences between the AIH and control groups and between overlap and control groups are significant for all cases, *p* < 0.01.

Circulating serum cytokine levels were examined in all AILD patients and were compared to the following levels of healthy controls. Overall, patients with both AIH and overlap syndrome, as well as the whole combined group of AILD, had significantly higher levels (as compared to controls) of IL-6 (0.70 (range 0.17–99.86) in patients with AILD compared to 0.40 (range 0.14–2.65) in controls, *p* < 0.01), IL-8 (1.66 (0.45–34.58) versus 0.53 (0.35–2.38), resp., *p* < 0.01), and TNF-*α* (2.61 (0.23–120.88) versus 1.65 (0.21–7.54), resp., *p* < 0.01). Distributions of IL-6, IL-8, and TNF-*α* levels are shown in Figure 1. In the group of patients with overlap syndrome, as well as in a general group of AILD, but not in patients with AIH, levels of IL-2 were significantly lower compared to controls (2.07 (range 0.11–111.87) in patients with AILD compared to 2.76 (range 0.45–4.10); *p* < 0.05). There were no differences detected in serum levels of other cytokines compared to control and in levels of all examined cytokines between the groups of AIH and overlap syndrome. Full data are shown in Table 2.

Revealed statistically significant associations were further analyzed by a regression analysis. Unadjusted logistic regression confirmed that the serum levels of IL-6, IL-8, and TNF-*α* were significantly higher in AILD patients compared to controls. This association was especially pronounced for IL-8 (odds ratio (OR) with 95% confidence interval (CI) – 57.54 (6.15–538.08)). Associations between IL-2 level and the disease were no longer significant. In subgroup analysis, OR for IL-8 were still high and statistically significant: 30.39 (3.15–293.12) for AIH and 22.12 (3.07–159.55) for overlap syndrome versus healthy controls. The combined adjusted regression model, which included all 4 cytokines and was adjusted for age and gender, also showed a pronounced marked relation of IL-8 and AILD, 48.36 (3.63–643.60), while the associations between the level of other cytokines and AILD were assessed as nonsignificant. In subgroup analysis, OR for IL-8 were still high and statistically significant after adjustment: 18.54 (1.08–318.54) for AIH and 23.85 (2.37–240.23) for overlap syndrome versus healthy controls. Full data are shown in Table 3.

### 3.3. Cytokine Levels and Their Association with Clinical and Laboratory Parameters

Parameters of liver damage, such as levels of liver enzymes, albumin, and bilirubin levels, and parameters of active ongoing inflammation, such as ESR and level of gamma-globulins, were explored for their association with circulating serum cytokine levels in whole group of AILD patients and in subgroups (AIH and overlap syndrome). The majority of correlations were observed for the level of IL-8, which has positively correlated with the level of ESR (*r* = 0.41; *p* = 0.002), AST (*r* = 0.32; *p* = 0.018), alkaline phosphatase (*r* = 0.45; *p* = 0.001), and total bilirubin (*r* = 0.42; *p* = 0.001) and negatively correlated with serum albumin level (*r* = −0.33; *p* = 0.026).  Table 4 gives all revealed correlations between serum cytokine levels and disease activity. No correlations were revealed for other laboratory or clinical parameters or serum cytokine levels.

## 4. Discussion

To our knowledge, this is the first study that compared the levels of a wide range of pro- and anti-inflammatory cytokines in patients with AILD, especially in patients with overlap syndrome, to healthy controls. In our study, serum levels of 4 cytokines, IL-2, IL-6, IL-8, and TNF-*α*, were significantly different in patients with AILD compared to healthy controls. The adjusted regression model has shown the central importance of IL-8, as this was the only cytokine, for which the difference between the patients with AILD and controls was still significant after adjustment. In the language of absolute numbers, the increase of IL-8 serum level by 1 pg/mL has increased the chance for a patient to find himself in a group of AILD by amazing 48.36 times, and in a group of overlap syndrome, by 23.85 times. Together with that, high IL-8 serum levels were strongly related to clinical parameters. Patients with AILD and higher serum IL-8 levels tended to have higher levels of total bilirubin, AST, alkaline phosphatase, and ESR and lower level of serum albumin, indicating lower liver synthetic function (albumin), higher grades of cytolysis (AST), and cholestasis (alkaline phosphatase), as well as higher grade of systemic inflammation (ESR). This association was almost lost in the subgroup analysis, which we hypothesize to be mainly due to a small sample size. Considering this, it seems that IL-8 levels might be of use for both establishing the diagnosis of AILD (i.e., as a diagnostic marker) and measuring the diseases activity and severity (i.e., as a clinical marker), which needs to be confirmed and explored in detail by further studies and supported by calculation of diagnostic utility parameters in future research.

Some other reports also showed significantly higher levels of all proinflammatory cytokines when compared to controls in patients with similar conditions. For example, a study in patients with primary biliary cirrhosis revealed the increased levels of IL-1*β*, IL-6, and TNF-*α* in such patients compared to healthy controls [12]. Another study revealed the possible association between the altered function of* T* cells and increased tissue level of TNF-*α* in patients with primary sclerosing cholangitis [13]. Considering the central pathogenetic role of Th17 cells in AIH, it is also important that Th17 cell-related gene expressions of such cytokines as IL-17, IL-23, IL-21, IL-1*β*, and IL-6 are reported to be significantly increased in the liver of AIH patients compared to healthy controls [14]. A study of cytokine levels in children with autoimmune hepatitis discovered a correlation between the levels of TNF-*α*, IL-6, and IL-8 and disease activity in patients with type 1 AIH. The levels of abovementioned cytokines were increased in children with active disease, as compared to controls, and decreased in patients with remission [15]. Our report, thus, confirms the hypothesis of a changed cytokine profile in patients with AILD, where the levels of proinflammatory cytokines, such as IL-6, IL-8, and TNF-*α*, would be increased compared to a healthy person, although the association between IL-8 serum level and AILDs, to the best of our knowledge, was never extensively studied, and such pronounced association was never reported before.

An elevation of IL-8 serum levels was shown before in patients with other liver diseases. Recently, a study of immune function in patients with cirrhosis has reported increased serum levels of proinflammatory cytokines, including IL-8. In this study, IL-8, together with IL-6, was higher in both patients with stable and those with decompensated cirrhosis and stayed high after development of acute-on-chronic liver failure [16]. It has been also shown that IL-8 levels are increased intrahepatically and in the serum of patients with alcoholic liver disease, probably contributing to hepatic neutrophil accumulation and also exerting systemic actions [17]. In patients with nonalcoholic fatty liver disease, interleukin-8 expression was also increased [18].

Of interest, patients with overlap syndrome in our study cohort had more severe and more rapidly progressing disease according to the clinical and laboratory parameters, with shorter disease duration, but the same frequency of severe fibrosis and higher levels of ALT, ESR, and alkaline phosphatase, compared to patients with isolated AIH. This was not an expected finding. It has been showed previously that AIH patients are significantly more likely to present with jaundice and have higher AST levels compared to patients with overlap syndromes [19]. This discrepancy might be partially explained by the age difference of nearly 10 years between the groups (nevertheless not statistically significant) and by lower percentage of patients with overlap syndrome receiving systemic treatment, such as glucocorticoids and cytostatics, at the time of inclusion.

Despite the difference in clinical parameters, the difference in IL-6 levels with the controls was somehow more marked in patients with AIH and the whole group of AILD, but not in patients with overlap syndrome. Nevertheless, in a subgroup of patients with overlap syndrome, but not in other patients, levels of IL-6 correlated with immunologic parameters, such as ESR and the serum level of *γ*-globulins. This comes in line with the differences of these two groups of patients. It is known that an autoimmune response is more pronounced in patients with overlap syndrome, leading to a higher production of autoimmune antibodies and to significant reduction of expected life duration [19]. IL-6, as the key cytokine mediating systemic immune response [20], may play a role in that.

## 5. Conclusions

In patients with AILD, cytokine profile significantly differs from that in healthy subjects, with increased serum levels of proinflammatory cytokines, such as IL-6, IL-8, and TNF-*α*. IL-8 seems to have the strongest relation to the disease and also correlates with the severity of laboratory abnormalities in patients with AILD. Possible use of IL-8 as a potential diagnostic or clinical marker, or a treatment target, is still to be explored.

## Figures and Tables

**Table 1:** Patients' characteristics.

	AILD (*n* = 60)	AIH (*n* = 32)	Overlap syndrome (*n* = 28)	*p*
Age, years (M ± SD)	48.2 ± 15.1	43.2 ± 15.5	53.9 ± 12.6	*p* > 0.05
Gender, females (%)	54 (90%)	28 (87.5%)	26 (92.8%)	*p* > 0.05
Symptom duration, years (M ± SD)	5.9 ± 5.6	6.9 ± 6.3	4.7 ± 4.5	**p** = 0.02
Jaundice (%)	36 (60%)	17 (53.1%)	19 (67.9%)	*p* > 0.05
Abdominal discomfort (%)	34 (56.7%)	16 (50%)	18 (64.3%)	*p* > 0.05
Extrahepatic signs (%)	41 (68.3%)	23 (71.9%)	17 (60.7%)	*p* > 0.05
Cirrhosis (%)	43 (71.7%)	23 (71.9%)	20 (71.4%)	*p* > 0.05
Hemoglobin, g/L (M ± SD)	120.9 ± 15.1	118.1 ± 20.2	124.2 ± 18.3	*p* > 0.05
ESR, mm/h (M ± SD)	24.7 ± 14.9	17.3 ± 12.1	33.12 ± 13.3	**p** = 0.000
ALT, U/L (M ± SD)	107.6 ± 143.1	68.8 ± 72.8	147.8 ± 183.6	**p** = 0.047
AST, U/L (M ± SD)	84.4 ± 68.0	72.9 ± 68.4	96.7 ± 66.6	*p* > 0.05
Alkaline phosphatase, U/L (M ± SD)	220.4 ± 68.0	107.7 ± 71.4	337.8 ± 176.9	**p** = 0.000
GGT, U/L (M ± SD)	258.9 ± 296.1	134.3 ± 135.8	389.1 ± 359.9	*p* > 0.05
Total bilirubin, *μ*mol/L (M ± SD)	58.9 ± 100.5	59.6 ± 91.47	58.2 ± 111.0	*p* > 0.05
Total IgG, mg/mL (M ± SD)	18.2 ± 5.3	18.6 ± 5.6	17.8 ± 4.8	*p* > 0.05
Circulating immune complexes, U/L (M ± SD)	241.1 ± 165.3	205.2 ± 133.6	283.5 ± 190.8	*p* > 0.05
Albumin, g/L (M ± SD)	48.3 ± 5.3	47.9 ± 5.9	48.9 ± 4.3	*p* > 0.05
Gamma-globulin, g/L (M ± SD)	24.9 ± 5.7	26.1 ± 6.2	23.2 ± 4.5	*p* > 0.05
UDCA intake (%)	52 (86.7%)	25 (78.1%)	27 (96.4%)	**p** = 0.037
GC intake (%)	20 (33.3%)	25 (78.1%)	15 (53.6%)	**p** = 0.044
GC dose, mg/day (M ± SD)	17.0 ± 11.8	26.1 ± 6.2	17.8 ± 15.5	*p* > 0.05
AZA intake (%)	6 (10%)	5 (15.6%)	1 (3.6%)	*p* > 0.05

AILD: autoimmune liver disease; AIH: autoimmune hepatitis; ESR: erythrocyte sedimentation rate; ALT: alanine aminotransferase; AST: aspartate aminotransferase; GGT: gamma-glutamyl transpeptidase; UDCA: ursodeoxycholic acid; GC: glucocorticoids; AZA: azathioprine.

**Table 2:** Serum circulating levels of cytokines in patients with AILD and in healthy controls. Numbers are presented as median (range).

	IL-2, pg/mL	IL-4, pg/mL	IL-6, pg/mL	IL-8, pg/mL	IL-10, pg/mL	IFN-*γ*, IU/mL	TNF-*α*, pg/mL
AILD (*n* = 60)	2.07 (0.11–111.87)^a^	0.18 (0.11–1.25)	0.70 (0.17–99.86)^b^	1.66 (0.45–34.58)^b^	0.57 (0.11–951.96)	4.77 (0.59–493.96)	2.61 (0.23–120.88)^b^
AIH (*n* = 32)	2.33 (0.19–41.33)	0.18 (0.11–0.34)	1.14 (0.20–19.4)^b^	1.50 (0.45–34.58)^a^	0.62 (0.22–29.94)	4.60 (0.59–133.32)	2.38 (0.56–52.96)^a^
Overlap syndrome (*n* = 28)	1.97 (0.11–111.87)^a^	0.18 (0.12–1.25)	0.60 (0.17–99.86)^a^	2.15 (0.60–27.60)^b^	0.52 (0.11–951.96)	5.12 (1.54–493.96)	2.66 (0.23–120.88)^b^
Control (*n* = 21)	2.76 (0.45–4.10)	0.17 (0.11–0.53)	0.40 (0.14–2.65)	0.53 (0.35–2.38)	0.42 (0.13–51.67)	5.65 (0.59–26.22)	1.65 (0.21–7.54)

AILD: autoimmune liver disease; AIH: autoimmune hepatitis; IL: interleukin; IFN-*γ*: interferon-gamma; TNF-*α*: tumour necrosis factor-alpha.

^a^ *p* < 0.05 compared with healthy controls group (Mann–Whitney *U* test);

^b^ *p* < 0.01 compared with healthy controls group (Mann–Whitney *U* test).

**Table 3:** Association (represented as odds ratios and 95% confidence intervals from the logistic regression analysis) between serum levels of cytokines (IL-2, IL-6, IL-8, and IL-10) and autoimmune liver diseases.

Parameter	Unadjusted model (all parameters analyzed separately)	Adjusted^*∗*^ combined model
*AILD*		
IL-2	1.01 (0.95–1.08)	0.58 (0.32–1.03)
IL-6	3.86 (1.04–14.41)	1.56 (0.75–3.25)
IL-8	57.54 (6.15–538.08)	*48.36 (3.63–643.60)*
TNF-*α*	1.55 (1.06–2.36)	1.37 (0.93–2.00)
Age	—	0.99 (0.94–1.05)
Gender (male)	—	3.13 (0.28–35.58)

*AIH*		
IL-2	1.03 (0.91–1.17)	0.65 (0.31–1.37)
IL-6	5.04 (1.25–20.32)	1.90 (0.70–5.13)
IL-8	30.39 (3.15–293.12)	*18.54 (1.08–318.54)*
TNF-*α*	1.41 (0.99–2.01)	1.35 (0.91–2.02)
Age	—	0.99 (0.92–1.05)
Gender (male)	—	1.59 (0.10–24.98)

*Overlap syndrome*		
IL-2	1.02 (0.96–1.08)	0.53 (0.26–1.08)
IL-6	2.17 (0.81–5.59)	1.68 (0.53–5.30)
IL-8	22.12 (3.07–159.55)	*23.85 (2.37–240.23)*
TNF-*α*	1.35 (0.88–2.08)	1.17 (0.58–2.38)
Age	—	1.02 (0.93–1.12)
Gender (male)	—	7.47 (0.29–192.75)

^*∗*^The model adjusted for the levels of IL-2, IL-6, IL-8, and TNF-*α*, age, and male gender. AILD: autoimmune liver disease; AIH: autoimmune hepatitis; IL: interleukin; IFN-*γ*: interferon-gamma; TNF-*α*: tumour necrosis factor-alpha.

**Table 4:** Spearman rank correlations between laboratory variables and serum cytokine levels measured. Numbers given are *r*(*p*) values. Statistically significant correlations are marked with italic font.

	Total bilirubin	ESR	AST	AlkP	Albumin	*γ*-Globulin
*AILD*						
IL-2	*−0.28 (0.034)*	0.03 (0.84)	−0.17 (0.21)	−0.16 (0.27)	0.07 (0.67)	0.09 (0.57)
IL-4	−0.04 (0.75)	0.16 (0.25)	−0.04 (0.80)	0.07 (0.65)	0.14 (0.37)	−0.04 (0.80)
IL-6	0.23 (0.08)	0.10 (0.46)	0.02 (0.88)	−0.12 (0.43)	*−0.30 (0.044)*	0.25 (0.10)
IL-8	*0.42 (0.001)*	*0.41 (0.002)*	*0.32 (0.018)*	*0.45 (0.001)*	*−0.33 (0.026)*	0.18 (0.23)
TNF-*α*	0.18 (0.17)	−0.07 (0.59)	0.11 (0.44)	0.12 (0.40)	−0.13 (0.41)	0.08 (0.61)

*AIH*						
IL-2	−0.25 (0.18)	−0.13 (0.50)	−0.37 (0.052)	−0.18 (0.39)	0.13 (0.53)	−0.06 (0.79)
IL-4	−0.21 (0.27)	−0.14 (0.47)	−0.14 (0.48)	*−0.45 (0.023)*	0.26 (0.20)	−0.07 (0.72)
IL-6	0.24 (0.20)	0.02 (0.90)	0.20 (0.30)	0.22 (0.29)	−0.29 (0.15)	0.02 (0.92)
IL-8	*0.43 (0.017)*	0.32 (0.09)	*0.40 (0.032)*	0.37 (0.07)	−0.32 (0.11)	0.20 (0.32)
TNF-*α*	−0.09 (0.63)	−0.36 (0.05)	0.05 (0.79)	0.03 (0.90)	−0.14 (0.50)	0.15 (0.47)

*Overlap syndrome*						
IL-2	−0.33 (0.08)	0.38 (0.07)	−0.03 (0.87)	−0.19 (0.38)	0.05 (0.86)	0.11 (0.65)
IL-4	0.21 (0.28)	0.31 (0.14)	−0.07 (0.74)	−0.01 (0.97)	−0.12 (0.62)	0.20 (0.41)
IL-6	0.27 (0.16)	*0.49 (0.014)*	−0.09 (0.67)	−0.20 (0.36)	−0.39 (0.10)	*0.46 (0.046)*
IL-8	0.34 (0.08)	0.27 (0.19)	0.09 (0.66)	0.19 (0.37)	−0.41 (0.08)	0.25 (0.31)
TNF-*α*	*0.56 (0.002)*	0.28 (0.17)	0.22 (0.27)	0.21 (0.32)	−0.12 (0.62)	0.09 (0.90)

AILD: autoimmune liver disease; AIH: autoimmune hepatitis; IL: interleukin; TNF-*α*: tumour necrosis factor-alpha; ESR: erythrocyte sedimentation rate; AST: aspartate aminotransferase; AlkP: alkaline phosphatase.
